# Water-Driven Oxygen and Nitric Oxide Release from Porphyrin- and Mn-Based Metal–Organic Framework Enables Accelerated Acute Wound Healing

**DOI:** 10.1021/acsabm.5c00135

**Published:** 2025-09-01

**Authors:** Jieh-Neng Wang, Zih-Yu Tu, Wen-Jyun Wang, Wei-Peng Li, Wei-Ling Chen, Chung-Dann Kan

**Affiliations:** † Department of Pediatrics, College of Medicine, 653089 National Cheng Kung University Hospital, Tainan 704, Taiwan; ‡ Department of Medicinal and Applied Chemistry, 38023 Kaohsiung Medical University, Kaohsiung 807, Taiwan; § Department of Medical Research, Kaohsiung Medical University Hospital, Kaohsiung 807, Taiwan; ∥ Drug Development and Value Creation Research Center, Kaohsiung Medical University, Kaohsiung 807, Taiwan; ⊥ Center of Applied Nanomedicine, National Cheng Kung University, Tainan 701, Taiwan; # Department of Biomedical Engineering, 46615 Taipei Veterans General Hospital, Taipei 112, Taiwan; ∇ Department of Biomedical Engineering, National Yang Ming Chiao Tung University, Taipei 112, Taiwan; ○ Division of Cardiovascular Surgery, Department of Surgery, College of Medicine, 34912 National Cheng Kung University Hospital, Tainan 704, Taiwan

**Keywords:** metal−organic framework, nitric oxide, porphyrin, gas therapy, wound healing

## Abstract

Medical gases, particularly oxygen and nitric oxide (NO), have attracted significant interest in clinical applications, notably wound healing, due to their role in enhancing cell proliferation, angiogenesis, and collagen deposition. However, the use of these gases has been limited by challenges such as inefficient gas delivery and potential toxicity to normal tissues. In this study, we elucidate a feasible approach using a porphyrin-based metal–organic framework (MOF), a unique material that shows immense potential for dual-gas-assisted wound healing. The MOF nanorods, meticulously designed, contain catalytically active manganese clusters, enabling spontaneous water decomposition and subsequent oxygen generation upon water exposure. The MOF incorporates Fe-chelated porphyrins, which not only serve as ligands connecting the manganese clusters but also exhibit a strong affinity with NO gas for the successful delivery of NO. The loaded NO, tethered to the MOF, can be released in a water environment. We employed a wound-healing assay to evaluate the efficacy of the NO-loaded MOF. After adding the MOF to fibroblast cell culture for O_2_ and NO supply, significantly accelerated migration and proliferation were obtained, providing strong evidence for the potential of the MOF in water-driven dual-gas therapy for wound care.

## Graphical abstract

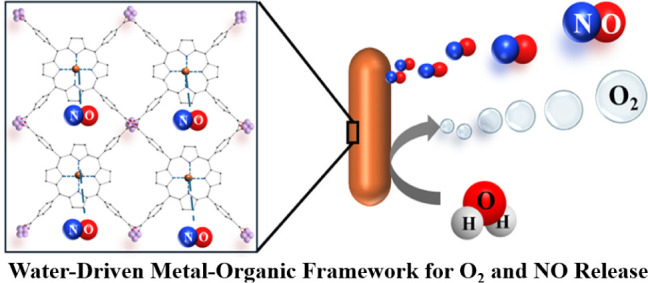

## 1 Introduction

Wound treatment stands as a critical aspect of healthcare. From superficial cuts to chronic ulcers, the spectrum of wounds demands tailored approaches to facilitate optimal healing and prevent complications.^1^ Traditional wound care methods focus on promoting healing, preventing infection, and minimizing complications associated with wounds.^2^ In general, wound healing is the body’s natural process of repairing damaged tissue, which occurs in four stages: hemostasis, inflammation, proliferation, and remodeling.^3^ In specific situations, chronic wounds often stall in one or more phases or fail to heal entirely within a typical time frame.^4^ Therefore, developing contemporary wound care methods by integrating traditional methods with advanced techniques and innovative materials to enhance chronic wound healing outcomes and minimize complications is a necessary practice and remains a big challenge.^5–7^ Notedly, the global wound care market has been consistently expanding, with its size projected to reach multibillion dollars due to the increasing prevalence of chronic wounds, such as diabetic ulcers and pressure sores.^8^

Gas therapy typically refers to the use of specific gases for therapeutic purposes in medicine.^9,10^ Some key types of gas therapies include oxygen (O_2_), nitric oxide (NO), and carbon dioxide (CO_2_) therapies,^11^ which have shown great benefits in enhancing wound healing. Adequate oxygen levels are vital for cells to produce energy through a process called cellular respiration, thus facilitating cell migration, proliferation, and the production of new tissue.^12^ O_2_ is also required for fibroblasts to produce collagen, a structural protein that provides strength and support to the healing tissue.^13^ Moreover, oxygen can boost the immune system’s ability to fight infection at the wound site.^14^ Oxygen-rich environments are often less favorable for certain types of bacteria, which can aid in controlling or eliminating infections.^15^ NO also serves as a signaling molecule to directly affect cell activity, thus regulating cell migration, proliferation, and tissue regeneration.^16^ NO plays a role in promoting vasodilation and angiogenesis, which can improve blood flow to the wound area, facilitating the delivery of oxygen and nutrients essential for healing.^17^ In addition, NO has antimicrobial properties, helping to combat certain types of bacteria and prevent infections at the wound site.^18^ Moreover, NO is also involved in the regulation of collagen synthesis.^19^ Using nanoparticles for medical gas delivery has been developed in recent years, offering opportunities to enhance biosafety, controllability, efficacy, and targetability in therapeutic processes.^20–23^ Our previous studies successfully demonstrated photoresponsive nanocarriers for NO and CO_2_ delivery to accelerate wound healing.^24,25^ In the new avenue of related development, nanoparticles can be applied to encapsulate multiple gases, allowing for synergistic or combination therapies for enhanced efficacy.^26,27^

Nanoscale metal–organic frameworks (MOFs) are innovative nanomaterials with unique properties that have gained attention for various applications, including medicine, environmental remediation, H_2_ storage, and catalysis.^28,29^ Some MOFs with enzyme-like catalytic activities were termed nanozymes.^30^ The porous structure of MOFs has demonstrated its potential in creating drug delivery systems (DDS) that transport chemotherapy drugs precisely to tumor sites, serving therapeutic purposes.^31^ Specifically, MOFs with manganese exhibit exceptional catalytic capabilities, oxidizing hydrogen peroxide into oxygen, thereby alleviating hypoxia in deep-seated tumors.^32,33^ The porous nature of the MOF underscores its potential to store and release gases, making it a promising carrier for gas therapy.^34,35^ Porphyrin, serving as a ligand for MOF (porphyrin-based MOF), utilizes its inherent photosensitive properties to generate reactive oxygen species (ROS) upon irradiation.^36,37^ Furthermore, with an iron center, porphyrin acts as a binder for NO, suggesting the potential of porphyrin-based MOF in delivering NO for treating cardiovascular diseases.^24,38^

The present study aims to utilize spindle-shaped porphyrin-based nanozymes to explore the potential of combined gas therapy in expediting wound healing. This nanozyme, built around manganese active sites, catalyzes water molecule oxidation, generating a sustainable supply of O_2_. Moreover, due to the natural affinity between NO molecules and porphyrin, the nanozyme can effectively carry NO. Under a water-based environment, significant NO gas release through spontaneous dissociation of the bond between NO and porphyrin was obtained (Scheme 1). Overall, this porphyrin-based nanozyme delivers substantial amounts of both O_2_ and NO, presenting promising synergistic effects of gas therapy for facilitating cell activation.

**Scheme 1:**
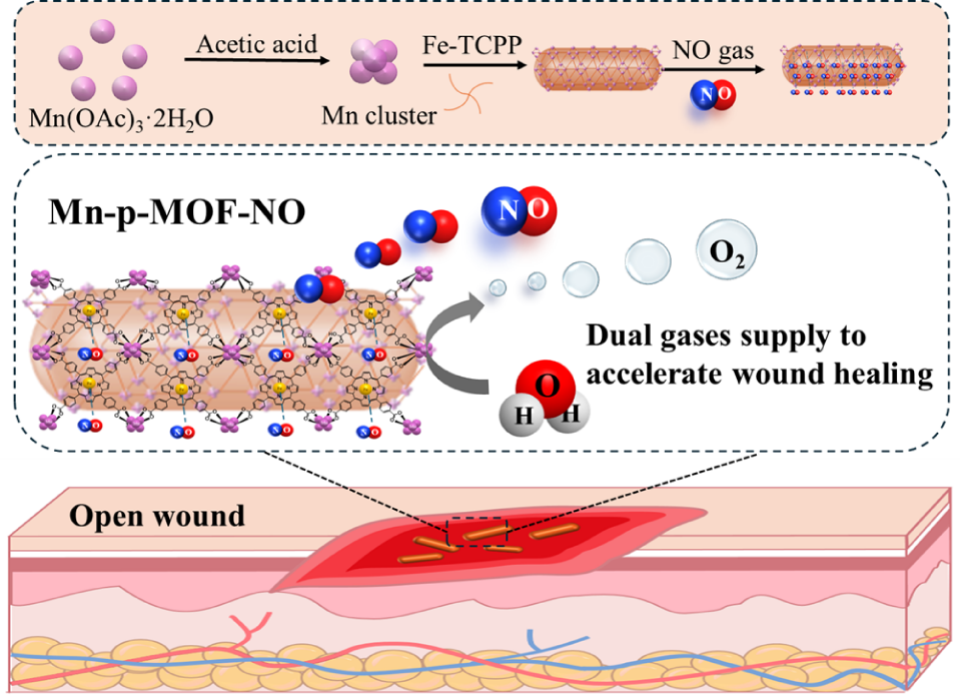
Cartoon Illustrates the Strategy of Combination Gas Therapy Using Water-Catalyzed Mn/Porphyrin-Based Nanozymes for Enhanced Wound Healing

## 2 Experimental Section

### 2.1 Materials

Dimethylformamide (DMF, 99.5%) was obtained from Duksan. Manganese­(III) acetate dihydrate (Mn­(OAc)_3_·2H_2_O, 97%), Ru­(dpp)_3_Cl_2_ (98%), and polyvinylpyrrolidone (PVP, average Mw = 55,000) were purchased from Sigma-Aldrich. Fe­(III) meso-tetra­(4-carboxyphenyl)­porphine chloride (Fe-TCPP, 95%) was obtained from Combi-Blocks. DAF-2 DA diacetate was purchased from AAT Bioquest. Acetic acid (99.7%) and hydrogen peroxide (H_2_O_2_, 30%) were obtained from SHOWA. Water purified using a Milli-Q Synergy system was used throughout the study.

### 2.2 Preparation of Mn/Porphyrin-Based MOF (Mn-p-MOF)

To begin, solution A was prepared by dissolving 5.6 mg of Mn­(OAc)_3_·2H_2_O and 5 mg of PVP in 2 mL of DMF. Solution B (8 mL) was modulated by mixing acetic acid and DMF at a volume ratio of 1:4. Solution B was then gently added to solution A while stirring for 5 min to form the Mn-cluster. Subsequently, 2.3 mg of Fe-TCPP dissolved in 2 mL DMF was slowly added dropwise into the Mn cluster solution under gentle stirring for 24 h to form the Mn-p-MOF. After that, the resulting solution underwent centrifugation at 7000 rpm for 5 min to collect the Mn/porphyrin-based MOF and eliminate residual reactants. Fresh DMF solvent was then added to disperse the Mn-p-MOF pellet, and this washing process was repeated at least three times. The Mn-p-MOF dispersed in DMF was stored at room temperature in the dark for use in subsequent experiments.

### 2.3 Preparation of NO-Loaded MOF

Due to the use of toxic NO gas, a detailed illustration of the operation flow of the NO-loading method is provided in Figure S1. The DMF with saturated NO dispersed in a sealed bottle was prepared by purging NO gas into 5 mL of DMF for 1 min. Then, the resulting DMF with saturated NO was diluted 10 times as the working NO-containing solution. A 0.15 mL of the working NO solution was added to a sealed bottle containing 15 mL of Mn-p-MOF nanozymes at 40 ppm of Fe element. This mixture was then left to stand for 30 min to yield NO-loaded MOF nanozymes (Mn-p-MOF-NO). Subsequently, the Mn-p-MOF-NO nanozymes were centrifuged at 7000 rpm for 5 min to obtain a Mn-p-MOF-NO pellet, followed by dispersion in fresh DMF. This washing process was repeated at least three times to ensure purity. The Mn-p-MOF-NO dispersed in DMF was stored at 4 °C in the dark for use in subsequent experiments.

### 2.4 Characterizations

The optical features of Mn-p-MOF nanozymes were measured by a UV–vis absorption spectrometer (Analytik Jena SPECORD 200 PLUS). Transmission electron microscopy (TEM, Hitachi H-7500) and scanning electron microscopy (SEM, Zeiss Auriga) were employed for morphology observation. High-resolution TEM with energy-dispersive X-ray spectroscopy (HR-TEM, JEOL JEM-2100F) was utilized for lattice image observation and elemental analysis. The dynamic light scattering spectrometer (DLS, Otsuka Electronics ELSZ-2000) was used to determine the zeta potential of Mn-p-MOF. Fourier transform infrared spectrometry (FTIR, Bruker Alpha 1) was applied to obtain the vibration spectra of Mn-p-MOF nanozymes. The crystalline phase of Mn-p-MOF was determined by X-ray diffraction (XRD, Bruker, D8 ADVANCE). The Fe concentration of the nanozymes was determined by atomic absorption (AA) spectroscopy. The binding energies of C, O, N, Mn, and Fe elements in the nanozymes were obtained by X-ray photoelectron spectroscopy (XPS, ULVAC-PHI, PHI Quantera II) analysis.

### 2.5 Evaluation of Oxygen Generation

To quantify the concentration of O_2_, we utilized the oxygen indicator Ru­(dpp)_3_Cl_2_ was utilized. Specifically, 1.5 mL of 4 μM Ru­(dpp)_3_Cl_2_ was introduced into 1.5 mL of deionized water. Subsequently, the solution was purged with N_2_ gas for 1 min to form an indicator stock solution stored in an inert environment. Following this, 3.1 mL of the aforementioned indicator solution was mixed with 0.2 mL of Mn-p-MOF and Mn-p-MOF-NO nanozyme solution, and 0.2 mL of 3% hydrogen peroxide. The concentration of nanozyme was fixed at 0.1 ppm Fe in the final solution. In the condition without H_2_O_2_, 0.2 mL of 3% hydrogen peroxide was replaced with 0.2 mL of deionized water. After a 10-min interval, the fluorescence intensity of Ru­(dpp)_3_Cl_2_ was measured using a fluorescence spectrometer (HORIBA, Fluoromax-4) to assess the oxygen generation triggered by the nanozyme-catalyzed water oxidation reaction. Notedly, the operation of the reaction and fluorescence measurement is in an open environment, causing unavoidable exogenous oxygen influence from the air. Therefore, a control group of indicators exposed to air has to be conducted for reasonable comparison with experiment groups to determine endogenous oxygen generation. The group of Mn-p-MOF without H_2_O_2_ was selected to evaluate time-dependent O_2_ production at 30, 60, and 90 min.

The hypoxia-inducible factor 1α (HIF-1α) antibody was applied as an indicator for cellular hypoxia labeling. Briefly, NIH/3T3 cells (4000 cells/well) were cultured, and then the Mn-p-MOF-NO at 0.1 ppm of Fe concentration were added to cell culture for 24 h-incubation in a 37 °C, 5% CO_2_ incubator. Afterward, the cells were stained with the indicator following standard staining protocols. The cell images were observed by a fluorescence microscope (Nikon ECLIPSE Ti2) with suitable fluorescence channels for visualizing the HIF-1α-specific indicators.

### 2.6 Quantitation of NO Release from Nanozymes

The quantification of the NO concentration in the sample solution was performed using the NO colorimetric assay kit (Elabscience). 0.3 mL of solutions containing Mn-p-MOF-NO nanozymes with various concentrations (0, 20, 40, 60, and 80 ppm at Fe) were prepared within the wells of a 96-well plate and then allowed to stand for 15 min. The solutions were centrifuged at 3100*g* for 10 min to isolate their supernatants. These supernatants were mixed with Griess reagent (a working solution within the NO colorimetric assay kit) for standard sample preparation according to the protocol. The NO amounts were determined by measuring the absorbance of Griess reagent at 550 nm using an ELISA reader (Biotek, Synergy HTX multimode). A calibration curve of absorbance versus NO amount was constructed by using the standard method provided by the assay kit. All measurements were conducted in quadruplicate.

### 2.7 MTT Cytotoxicity Assay

The cytotoxicity assay utilized a fibroblast cell line (NIH/3T3). These cells were cultured in F12K supplemented with 0.03 mg/mL ECGS, 0.1 mg/mL heparin, 1% penicillin/streptomycin, and 10% FBS in a 37 °C, 5% CO_2_ incubator. At a density of 4000 cells per well, they were seeded into a 96-well plate and incubated at 37 °C for 24 h. Subsequently, the cells in each well were washed thrice with PBS solution. Fresh medium containing nanozymes at varying concentrations (0, 0.001, 0.01, 0.1, 1, and 2 ppm of Fe element) was added, and the cells were further incubated for 24 h. Following this incubation period, the cells treated with nanozymes underwent washing with PBS solution three times, followed by the standard MTT staining protocol to assess and quantify cell viability.

### 2.8 *In Vitro* Wound Healing Assay

NIH/3T3 cells were cultured in a plate until they formed a confluent monolayer. Using a fine tip creates a straight-line scratch across the cell monolayer. The cells were washed with culture medium to remove any detached cells or debris. After that, nanozymes (Mn-p-MOF and Mn-p-MOF-NO) containing 0.1 ppm of Fe element were added to the cell cultures. The scratch images at regular intervals (0, 2, and 24 h) were observed by using a microscope (Nikon ECLIPSE Ti2). All measurements were conducted in quadruplicate.

### 2.9 Fluorescence Imaging Observation of Intracellular Gas Release from Nanozymes

The DAF-2 DA was applied as an indicator for intracellular NO labeling. Briefly, NIH/3T3 cells (4000 cells/well) were cultured and then stained with the indicator following their standard staining protocols. After that, Mn-p-MOF and Mn-p-MOF-NO at 0.1 ppm of Fe concentration were added to cell culture for 1 h incubation in a 37 °C, 5% CO_2_ incubator. Afterward, the cell images were observed by a fluorescence microscope (Nikon ECLIPSE Ti2) with suitable fluorescence channels for visualizing the NO-specific indicators.

### 2.10 *In Vivo* Study of the Efficacy of Nanozyme for Actual Wounds

All animal experiments were conducted using male C57BL/6 mice aged between 9 and 10 weeks. Animal care and use were approved by the Institutional Animal Care and Use Committee (IACUC) of Kaohsiung Medical University (IACUC NO.112100; approval date: 20231228) and the IACUC of National Cheng Kung University (IACUC NO.113155; approval date: 20240324). All animal treatments and surgical procedures were performed in accordance with the guidelines of the Center for Laboratory Animals at Kaohsiung Medical University and the Laboratory Animal Center at the National Cheng Kung University. Anesthesia was induced using isoflurane (3 mg/100g). Following anesthesia, the dorsal hair was removed, and a 10 mm diameter wound was created. The mice were categorized into three groups: control (PBS alone; *n* = 3), Mn-p-MOF (*n* = 3), and Mn-p-MOF-NO (*n* = 3). For treatment, each wound on anesthetized mice was treated with 10 μL of buffer with or without nanozymes (0.1 ppm of Fe element). All brown-colored nanozyme buffer remained entirely on the local wound area, and the humid wound became a dry wound 30 min after administration. Afterward, the mice woke up, and all of the MOF stayed at the local wound site. Moreover, no residual brown-colored MOF was observed on the local wound or scab site after 1 day past administration, thus speculating all nanozymes were wholly absorbed by the mice’s bodies . Compared to the circular wound model, the size of the elongated wound is larger, and pulling of the wound is more frequent due to the mice’s movements when getting up to drink water in the cage, thereby resulting in a more extended wound healing period and higher similarity to actual wound conditions.^24,25^ Therefore, this model is suitable and reliable for monitoring the difference in wound healing rates between the control and experimental groups.

### 2.11 Histochemistry

The skin tissues and main organs, including the heart, liver, spleen, lungs, and kidneys, were obtained from mice with and without Mn-p-MOF-NO treatment on day 0. These tissues were embedded in paraffin and cut into thin sections by using a microtome. Samples were deparaffinized with xylene and rehydrated through a series of graded ethanol solutions. Then, Masson’s trichrome staining was applied to skin tissue after 7 days of treatment, and all tissue sections were examined under a microscope.

### 2.12 Blood Biochemical Analysis

The blood samples were collected from the mice after wound treatment. The blood was centrifuged at 1200 rpm at 4 °C for 10 min to obtain the serum. Then, blood biochemical analysis of serum was performed using appropriate assay kits to evaluate markers related to liver (ALT, AST) and kidney (creatinine, BUN) function indexes.

### 2.13 Biodistribution Evaluation

Mice were sacrificed at predetermined time points (day 0) after nanozyme treatment on the wound. The major tissues, including the heart, liver, spleen, lungs, and kidneys, were collected to evaluate the accumulation of nanozymes. These tissues were broken down using a homogenizer and then dispersed in aqua regia for 7 days to release Fe ions from nanozymes. The Fe concentration in each tissue was determined by AA.

## 3 Results and Discussion

### 3.1 Characterization of Mn/Porphyrin-Based MOF

In the present study, the catalytic Mn-p-MOF nanocrystal containing Mn clusters and porphyrin ligands (Mn-p-MOF) was successfully prepared, referring to a reported approach.^39^ The morphology of Mn-p-MOF was observed by TEM and SEM imaging, showing monodispersed nanorods with particle sizes of 306.7 ± 8.2 nm in length and 40.0 ± 4.0 nm in width, respectively (Figure 1a,b). The Mn-p-MOF colloidal solution showed a brown color without any aggregates. A high-resolution TEM image of a Mn-p-MOF nanorod revealed a well-defined rod shape and lattice growth toward the [100] direction (Figure 1c). The elemental analysis of Mn-p-MOF showed homogeneous Mn and Fe distribution within a particle, implying a structural composition containing Mn metal clusters and porphyrin ligands (Figure 1d). Additional XPS analysis also indicated the presence of Mn, Fe, N, and O elements in the Mn-p-MOF nanocrystal constructed by Fe-chelated porphyrin and a Mn-embedded acetic acid cage (Figure S2). The X-ray diffractometer (XRD) analysis of Mn-p-MOF indicated a characteristic profile of diffraction peaks, demonstrating the formation of a porphyrin-based Mn MOF crystal (Figure 1e).^39^ Furthermore, small-angle diffraction signals were also detected, suggesting the presence of intrinsic microstructures in the Mn-p-MOF, a typical structural feature observed in various MOFs.

**Figure 1:**
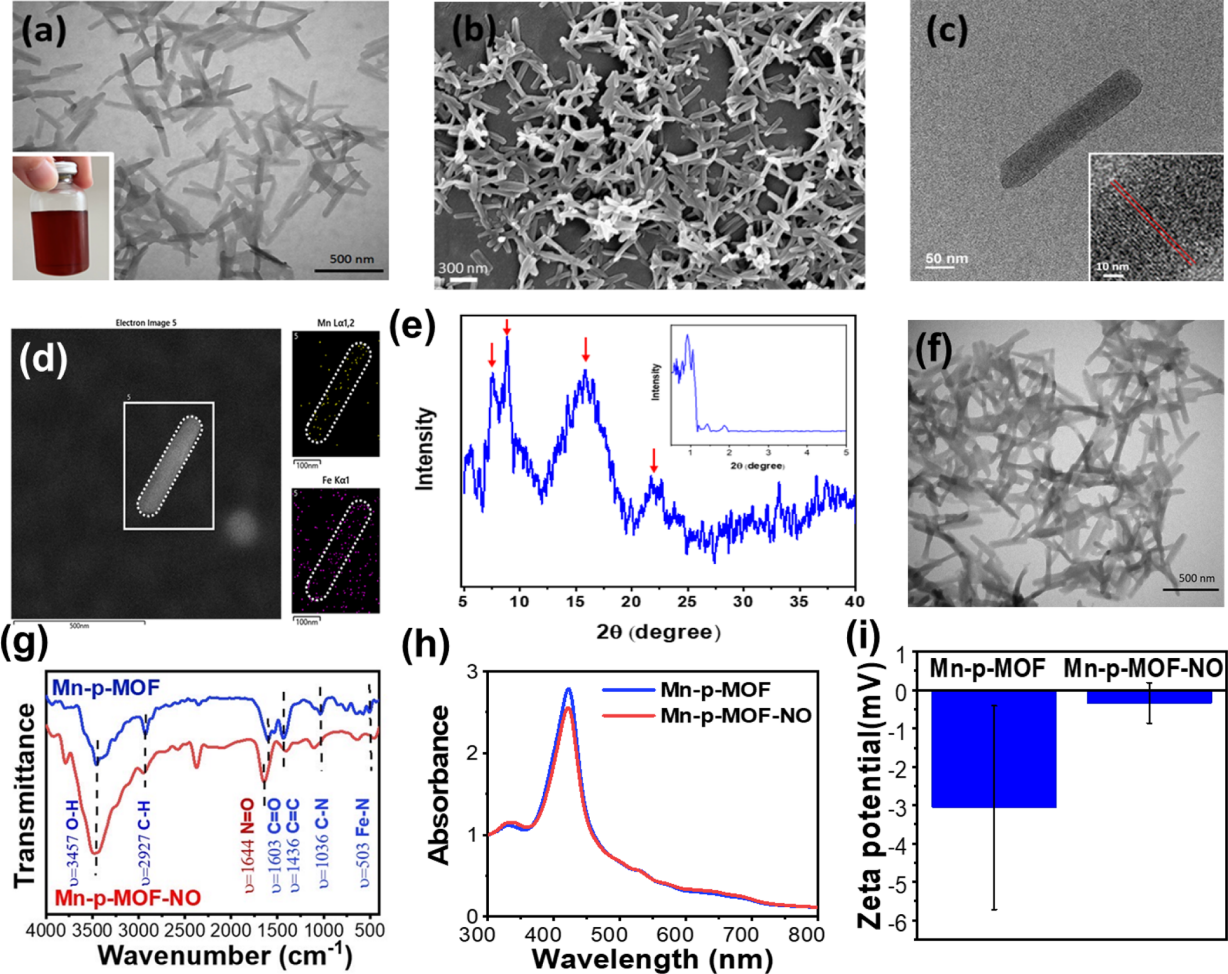
The characterization of Mn-p-MOF before and after NO loading. (a) TEM and (b) SEM images of Mn-p-MOF nanozymes. The inset photo in (a) shows the colloidal solution of Mn-p-MOF nanozymes. (c) The high-resolution TEM image of the nanozyme. The red lines in the inset image indicate the [110] lattice plane in the spindle-shaped Mn-p-MOF nanozyme. (d) The additional elemental analysis shows the Mn and Fe distribution in a selected area (white frame) with a nanozyme (white dashed line). (e) The XRD pattern of Mn-p-MOF nanozyme. The red arrows indicate representative peaks of Mn-p-MOF, whose peak positions are the same as the XRD results of the porphyrin and Mn-based MOF.^39^ The inset figure shows the small-angle analysis of Mn-p-MOF. (f) TEM image of Mn-p-MOF-NO nanozymes. (g) FTIR, (h) UV–vis spectra, and (i) zeta potentials of the MOF nanozymes before and after NO loading.

The Mn-p-MOF, composed of Mn clusters and Fe-doped TCPP ligands, inherently possesses porous characteristics with a high surface-to-volume ratio, making it an ideal cargo carrier with excellent loading efficiency. Furthermore, the TCPP, comprising a porphyrin moiety with a central Fe ion, exhibits strong affinity for NO through axial coordination between NO and Fe, endowing the capability of NO loading to Mn-p-MOF.^40,41^ We believe this chelation strategy for NO loading on porphyrin-based MOFs significantly enhances NO deposition and stability compared with gas donor loading on traditional MOFs, which rely on electrostatic interactions for gas adsorption. Therefore, the Mn-p-MOF was immersed in the NO-containing solution, resulting in the coordination between NO and porphyrin and the generation of the NO-loaded MOF nanorod (Mn-p-MOF-NO). No visible morphology or size changes of the MOF were observed after NO loading (Figure 1f). Interestingly, clear lattice patterns of the MOF framework were observed before NO loading, whereas these lattice patterns disappeared following NO loading, suggesting possible coverage of the MOF crystal surface by deposited NO molecules (Figure S3). In addition, the dark-field TEM images of Mn-p-MOF and Mn-p-MOF-NO also revealed obvious differences (Figure S4). Specifically, many bright white spots appeared on the MOF rods after NO loading, indicating that NO molecules grafted onto the TCPP-chelated Fe sites significantly affect the localized structure of the MOF crystals. Moreover, Fourier-transform infrared spectroscopy (FTIR) was applied to detect the vibration signals of the surface functional groups on Mn-p-MOF and Mn-p-MOF-NO (Figure 1g). The FTIR spectrum of Mn-p-MOF shows representative peaks at 503, 1036, 1436, 1603, 2927, and 3457 cm^–1^, corresponding to vibration models of Fe–N, C–N, C=C, C=O, C–H, and O–H bonds presented in the MOF constructed with Fe-TCPP and acetate acid. In the FTIR spectrum of Mn-p-MOF-NO, a significantly amplified vibration peak at 1644 cm^–1^ was obtained, which reflects the presence of the C=O-overlapped N=O signal and evidences the successful loading of NO on MOF. We did not find a considerable difference in the absorbance spectra of MOF nanorods before and after NO loading, indicating the excellent stability of Mn-p-MOF (Figure 1h). The surface negative charge of the MOF was neutralized after NO loading (Figure 1i). All the characterization results indicate the successful fabrication of Mn-p-MOF-NO.

### 3.2 Water-Driven O_2_ Release from Catalytic MOF

Mn-p-MOF contained Mn-based active sites on the surface; thus, it was predicted to have catalytic function for water oxidation and oxygen generation.^42,43^ Here, fluorescent Ru­(dpp)_3_Cl_2_ (Ru dye) was applied as the oxygen indicator for the oxygen production evaluation. Notedly, its fluorescence intensity at 613 nm shows an inverse relationship with the surrounding oxygen concentration. Interestingly, a significant intensity decrease of the Ru dye was observed in the presence of Mn-p-MOF, echoing the proposed catalytic ability of the Mn active site in turning water into O_2_ (Figure 2a). A slight decrease in oxygen production from Mn-p-MOF-NO indicated spatial hindrance by NO, inhibiting the adhesion of water to the Mn-exposed surface. Moreover, no enhanced oxygen production was found when the highly active oxidant hydrogen peroxide was added to the system, indicating that the presence of the oxidant might inhibit the catalytic performance of Mn-p-MOF. The lack of significant difference in fluorescence intensity over time implies that a rapid dynamic balance is reached in the water oxidation reaction activated by catalytic Mn-p-MOF (Figure 2b). Additionally, cellular assays involving staining for HIF-1α were performed (Figure S5).^44^ As anticipated, cells treated with Mn-p-MOF-NO showed notably reduced HIF-1α expression compared with untreated cells, supporting the conclusion that intracellular oxygen production by the nanozyme effectively mitigated mild hypoxic conditions. Additional XPS analysis was applied to further investigate the changes in Mn-p-MOF before and after the water oxidation reaction (Figure 2c–f). The ratios of Fe^2+^/Fe^3+^ and Mn^3+^/Mn^4+^ were determined through the volume ratio of the corresponding split peaks. Interestingly, a significant decrease in the ratio of Mn^3+^/Mn^4+^ from 3.2 to 0.72 was observed after immersing Mn-p-MOF in water, correlating the fact that manganese clusters serve as the catalytically active center to activate water decomposition upon water exposure. No considerable change in the ratio of Fe^2+^/Fe^3+^ after the water-driven oxygen generation reaction was observed, indicating no correlation of the iron-chelated ligand in oxygen evolution. The binding energies (C, O, and N) of Mn-p-MOF nanozymes in DMF and H_2_O are shown in Figure S6. The results reveal a significantly increased binding energy of Mn–O from 529.6 to 530.2 eV and a constant binding energy of Fe–N upon water exposure, attributed to the stronger coordination of the carboxyl ligand to electron-deficient Mn^4+^ than Mn^3+^, which repeatedly evidences the traces of the redox reaction in the catalytic Mn cluster.

**Figure 2:**
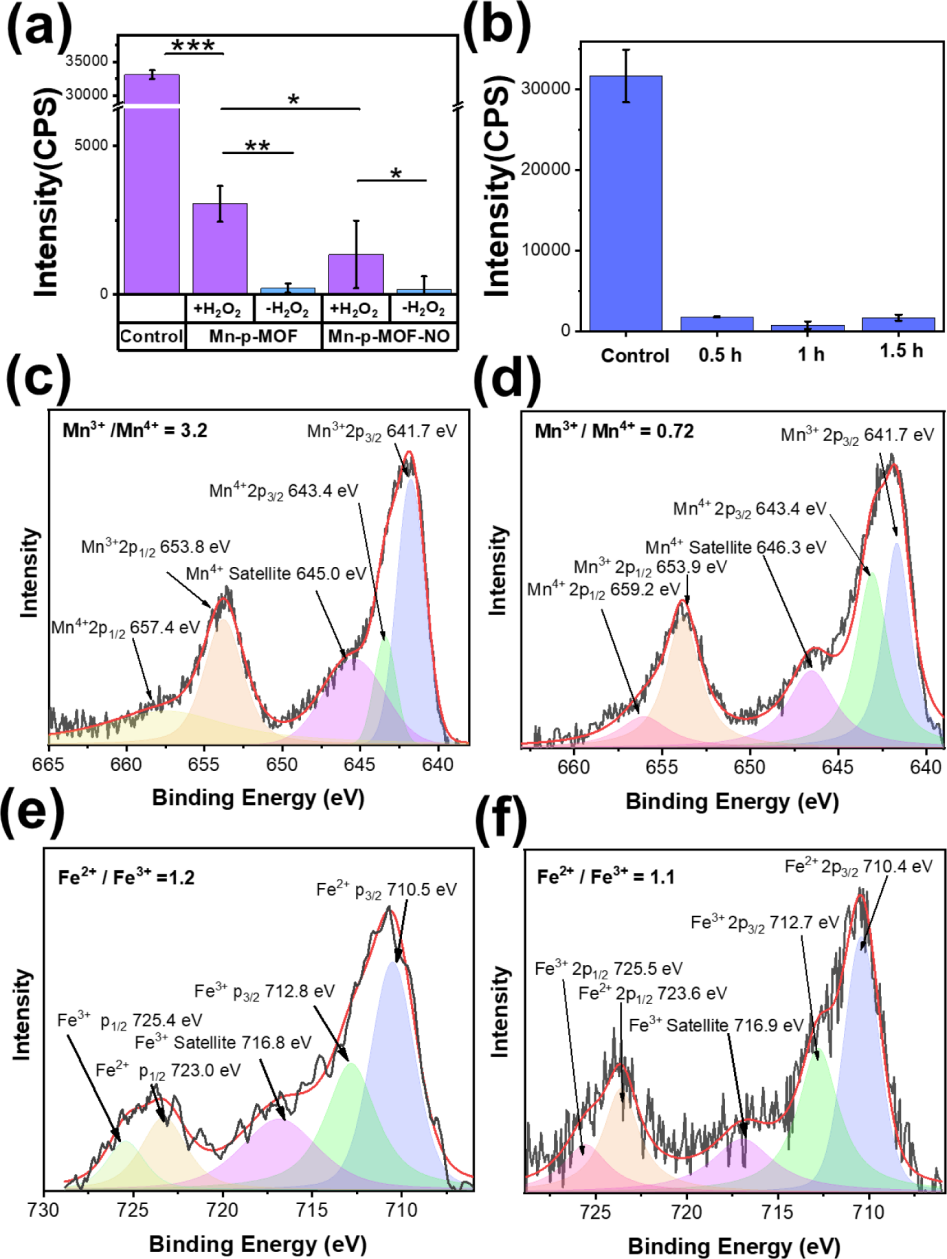
The evaluation of O_2_ generation from MOF. (a) Detecting the O_2_ amounts under different conditions (control, Mn-p-MOF nanozyme, and Mn-p-MOF-NO nanozyme at 0.1 ppm of Fe element with and without hydrogen peroxide) using Ru dye and fluorescence measurement after 10 min of being dispersed in water. (b) The kinetic evaluation of MOF-catalytic water oxidation. All measurements were conducted in quadruplicate. (c) For the Mn-p-MOF nanozymes in dimethylformamide (DMF), the binding energies of Mn­(III, 2P_3/2_), Mn­(IV, 2P_3/2_), Mn­(IV, Satellite), Mn­(III, 2P_1/2_), and Mn­(VI, 2P_1/2_) correspond to 641.7, 643.4, 645.0, 653.8, and 657.4 eV, respectively. (d) For the Mn-p-MOF nanozymes in H_2_O, the binding energies of Mn­(III, 2P_3/2_), Mn­(IV, 2P_3/2_), Mn­(IV, Satellite), Mn­(III, 2P_1/2_), and Mn­(VI, 2P_1/2_) correspond to 641.7, 643.4, 646.3, 653.9, and 659.2 eV, respectively. (e) For the Mn-p-MOF nanozymes in DMF, the binding energies of Fe­(II, 2P_3/2_), Fe­(III, 2P_3/2_), Fe­(III, Satellite), Fe­(II, 2P_1/2_), and Fe­(III, 2P_1/2_) correspond to 710.5, 712.8, 716.8, 723.0, and 725.4 eV, respectively. (f) For the Mn-p-MOF nanozymes in H_2_O, the binding energies of Fe­(II, 2P_3/2_), Fe­(III, 2P_3/2_), Fe­(III, Satellite), Fe­(II, 2P_1/2_), and Fe­(III, 2P_1/2_) correspond to 710.4, 712.7, 716.9, 723.6, and 725.5 eV, respectively. (*p < 0.05; **p < 0.01; ***p < 0.001).

### 3.3 NO Supply from Mn-p-MOF-NO under a Water-Based Environment

On the other hand, the Griess assay was applied to determine the NO level in the closed systems. A calibration curve shows a highly linear relationship between the NO concentration and the absorbance of the Griess reagent, enabling the subsequent quantification analysis of NO gas released from the MOF (Figure 3a). Interestingly, a tendency to increase the NO level as a function of increased Mn-p-MOF-NO concentration was obtained, thus indicating the feasible strategy of spontaneous NO release from the MOF carrier (Figure 3b). Under the water-based environment, the NO coordination bond on the axial position of the porphyrin ligand can be replaced with surrounding water molecules, thus liberating the NO from MOF carrier spontaneously.^45,46^ Moreover, the NIH/3T3 cell, a fibroblast cell line, was applied as the cell model to evaluate the capability of NO release of Mn-p-MOF-NO inside the cell. The DAF-2 DA dye was used as the intracellular NO indicator. Interestingly, significant green fluorescence emission of DAF-2 DA dye was observed in the group of Mn-p-MOF-NO to indicate the high amount of NO released inside the cell (Figure 3c). No visible signal was obtained in the control and Mn-p-MOF groups.

**Figure 3:**
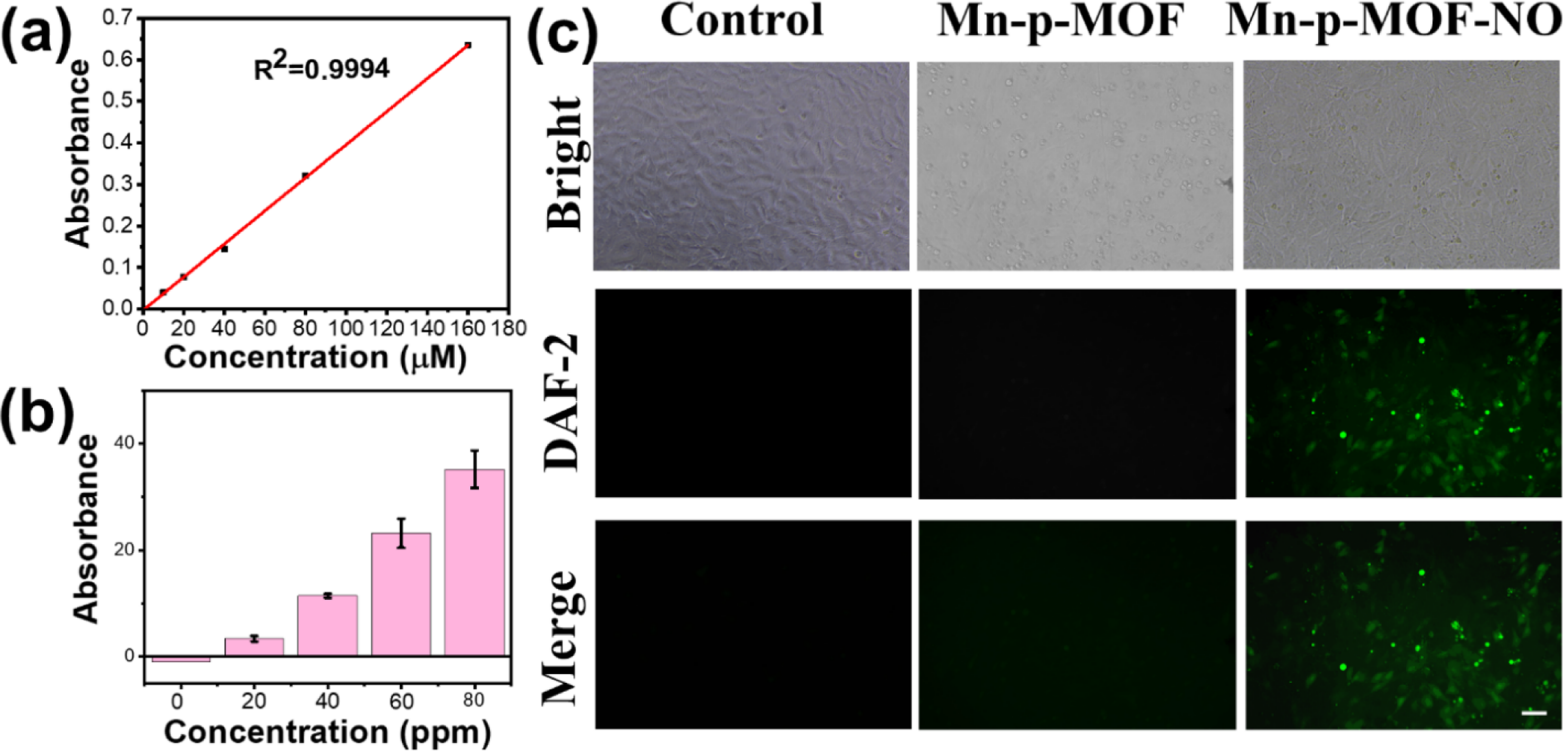
Evaluation of NO supply from Mn-p-MOF-NO. (a) Calibration curve of absorbance of Griess reagent at 550 nm vs known NO amounts. (b) The evaluation of NO release from Mn-p-MOF-NO nanozyme at different concentrations (0, 20, 40, 60, and 80 ppm of Fe element) dispersed in water for 15 min. All measurements were performed in triplicate. (c) Cell images of DAF-DA-stained NIH/3T3 cells incubated with and without Mn-p-MOF and Mn-p-MOF-NO nanozymes at 0.1 ppm Fe for 1 h. The green fluorescence signal indicates the presence of intracellular NO gas, visualized using a regular FITC channel of a microscope. The scale bar is 50 μm.

### 3.4 Wound Healing Assay of Mn-p-MOF-NO

The MTT assay was applied to evaluate the biocompatibility of the nanozyme to NIH/3T3 cells, which are a type of connective tissue cell commonly used for wound healing and tissue remodeling studies.^47,48^ No considerable cytotoxicity of NIH/3T3 cells treated with Mn-p-MOF and Mn-p-MOF-NO nanozymes was obtained, reflecting the excellent biocompatibility of nanozymes (Figure 4a). Notedly, the interesting enhancement of cell proliferation was obtained upon incubating with Mn-p-MOF from 0.01 to 1 ppm of Fe concentration and Mn-p-MOF-NO from 0.001 to 0.1 ppm of Fe concentration, implying the self-oxygen supply from nanozymes to activate the cell activity. The lower requirement of concentration range for cell activation in Mn-p-MOF-NO condition also implies the additional function of NO in enhanced cell proliferation. Moreover, a standard wound healing assay was applied to evaluate the potential efficacy of Mn-p-MOF-NO for accelerating wound repair (Figure 4b). Under observation with an optical microscope, the NIH/3T3 cell distribution shows a consistent gap to mimic the wound area. Interestingly, a significant decrease in the distance of the gap was observed in the group of Mn-p-MOF-NO for 2 h incubation, and the wounds were completely closed after 24 h incubation, implying an incredible efficacy of dual gas supply for facilitating cell migration and growth (Figure 4c). Under the single gas condition (Mn-p-MOF), the wound closure rate is significantly slower compared to dual gas treatment, but it also shows considerable cell activation compared to the nongas group (control), implying that the oxygen supply from nanozyme is an essential factor in accelerating cell growth. After 24 h, a relatively high cell density was obtained in the dual gas therapy group, presenting the innate function of medical gas to activate cell proliferation (Figure 4d).

**Figure 4:**
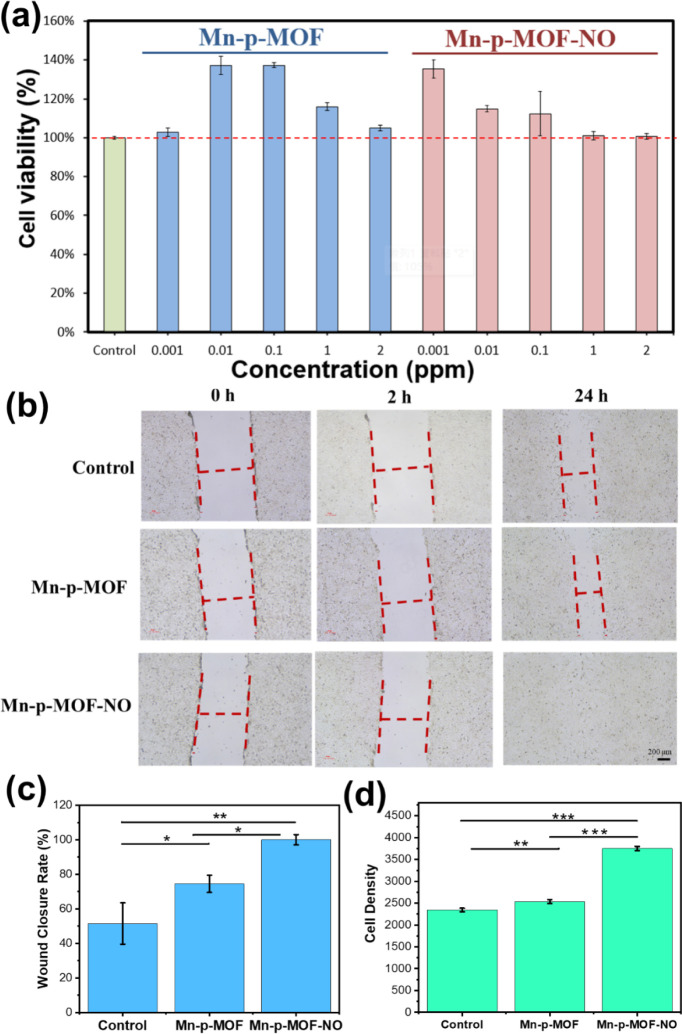
*In vitro*studies of Mn-p-MOF-NO nanozymes. (a) MTT assay of NIH/3T3 cells treated with Mn-p-MOF and Mn-p-MOF-NO nanozymes at different concentrations (0, 0.001, 0.01, 0.1, 1, and 2 ppm of Fe element). (b) The NIH/3T3 cell-based wound healing assay of Mn-p-MOF and Mn-p-MOF-NO nanozymes at 0.1 ppm Fe concentration for 0, 2, and 24 h incubation. The red dashed lines mark the edge and distances of cell clusters. (c) The wound closure rate was determined by the ratio of wound widths at 0 and 24 h. (d) The cell density was counted from the whole cell image at 24 h. All measurements were performed in triplicate. (*p < 0.05; **p < 0.01; ***p < 0.001).

### 3.5 Evaluation of Dual Gas Therapy in an Acute Wound Model

The mice with an elongated acute wound of 1 cm on the back were used to evaluate the feasibility and biosafety of the nanozymes (Figure 5a). The nanozyme pellet was dispersed in deionized water for activation to gradually release O_2_ and NO. The activated nanozymes were immediately applied to the wound for treatment. Three dosages of nanozymes were administered on days 0, 1, and 4. Under the monitoring of wound closure from days 1 to 7, the results demonstrated no significant difference between the control and Mn-p-MOF groups, suggesting that the O_2_ supply alone did not noticeably enhance wound healing (Figure 5b,c). However, treatment with the dual gas-supplying Mn-p-MOF-NO resulted in significantly accelerated wound closure, indicating that the dual-gas strategy was substantially more effective compared to single-gas treatment. Additionally, H&E and Masson’s trichrome staining showed complete epidermal repair with abundant collagen deposition in the dermis layer after 7 days of dual-gas treatment, compared to the control and Mn-p-MOF groups (Figure 5d), echoing the result of the wound healing assay to indicate the excellent efficacy of dual gas therapy by the MOF carrier.

**Figure 5:**
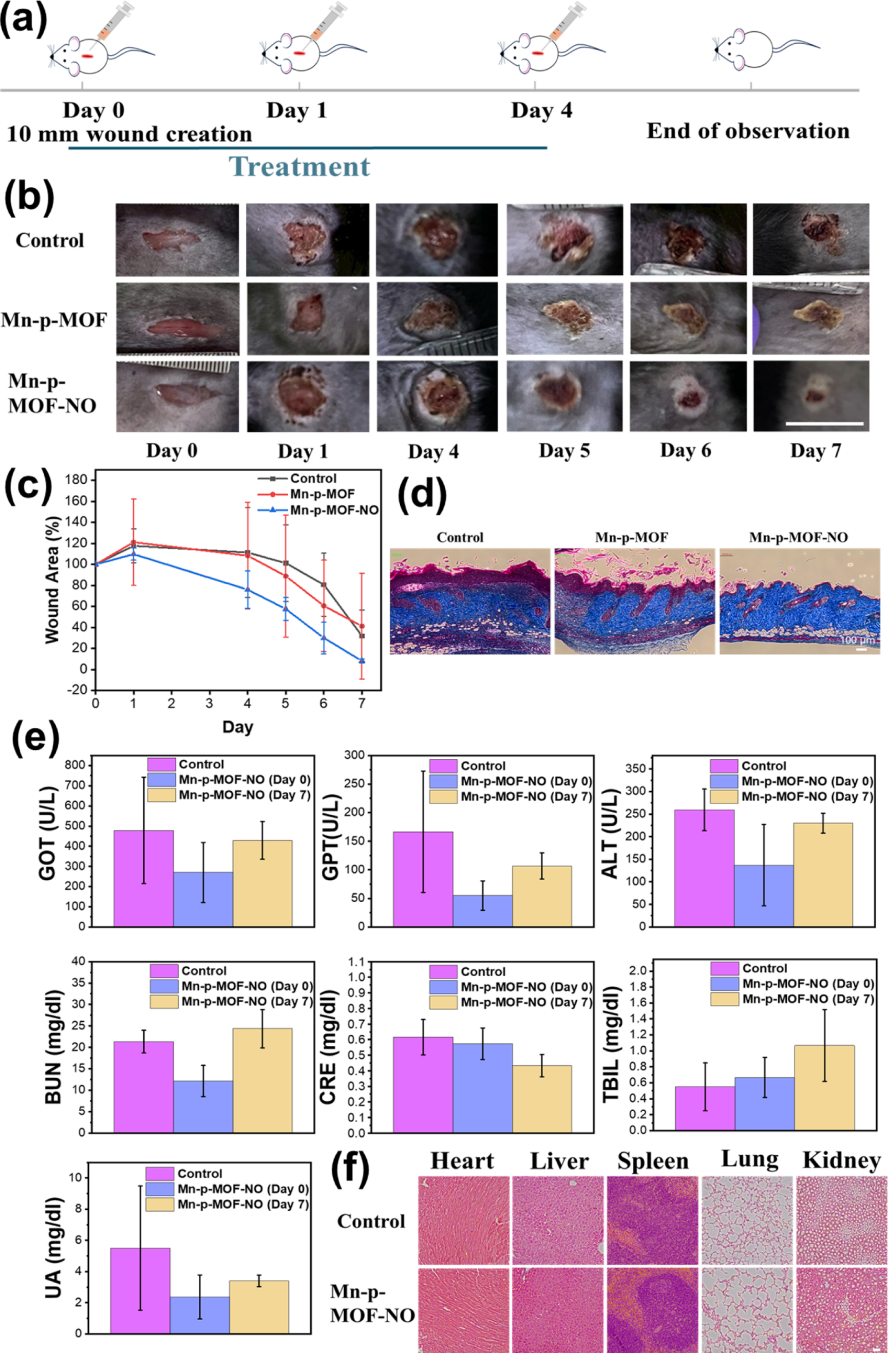
*In vivo* studies of Mn-p-MOF-NO nanozymes. (a) The cartoon illustrates the timeline of wound creation, treatment, and observation in the animal study. (b) Photos of wounds treated with and without nanozymes (Mn-p-MOF and Mn-p-MOF-NO). The scale bar is 10 mm. (c) Wound size observation for untreated and MOF-treated mice. (d) Images of Masson’s trichrome staining of skin tissue treated with and without nanozymes after 7 days. The scale bar is 100 μm. (e) Blood biochemical and (f) histochemistry analyses for mice treated with and without Mn-p-MOF-NO nanozymes at day 0. The scale bar is 100 μm.

Considering the nano scale of the Mn-p-MOF-NO crystal, the nanozyme can quickly penetrate the subcutaneous tissue *via* the open wound, thus either entering the systemic circulation or locating in the intercellular matrix. Even so, due to the smaller amount of dosage applied to the wound, no visible MOF residual was found in major organs (Figure S7). In the blood biochemical analysis of mice treated with Mn-p-MOF-NO after 0 and 7 days, no significant difference in liver and kidney indexes were measured compared to healthy mice, indicating the high biosafety of Mn-p-MOF-NO (Figure 5e). In the histochemistry analysis, no abnormal tissue changes in the main organs were observed in the treated mice (Figure 5f). Overall, the outcome of this preliminary animal study demonstrated the satisfactory efficacy and safety of dual gas therapy using Mn-p-MOF-NO, pointing to a promising direction in wound-care development.

For this dual gas delivery system, the concern about NO_2_ production is inevitable when NO and O_2_ coexist in a microenvironment (2NO + O_2_ → 2NO_2_), which must be discussed. Basically, the possible hazards to cells and wound tissue caused by cytotoxic NO_2_ are dependent on the concentration of NO_2_. In the wound treatment system, the maximum yield of NO_2_ can be determined by the concentration ratio of NO to O_2_ and their concentration levels, based on its stoichiometry. However, due to the inherent property of gas diffusion, precisely detecting gas levels in in vivo conditions can be challenging, as it is influenced by multiple factors, including biological environments, administration methods, and release rates. Therefore, in this study, we determined the treatment dosage based on the concentration of Mn-p-MOF-NO, which reveals the proportional relationship with NO and O_2_ supply.^49–51^ For future applications, the optimal dosage of Mn-p-MOF-NO with minimal NO_2_ toxicity can be further tailored according to specific disease conditions. Even so, NO_2_ concentration might be low based on the result of the MTT assay, which shows no significant cytotoxicity after NO and O_2_ cosupply to the NIH/3T3 cells (Figure 4a). Moreover, the trace NO_2_ might benefit wound healing due to the HNO_3_ and HNO_2_ production after NO_2_ is dissolved in water, thus giving the wound a weak acid environment and contributing to an additional Bohr effect.^25^ Additionally, the catalytic MOF might provide the unique ability to spontaneously decompose NO_2_ into N_2_ and O_2_, which can act as a last line of defense to efficiently adjust the actual wound condition if excess NO_2_ is generated.^52^ Based on these effects, we believe that Mn-p-MOF-NO is a reliable and safe dual gas system for wound care.

## 4 Conclusion

In the present study, the feasibility of synergistic medical gas therapy was evaluated using a catalytic MOF as the gas carrier. The oxygen supply from the catalytic Mn-based MOF to decompose the water into oxygen was achieved. The spontaneous NO release from the Mn-p-MOF-NO nanozyme was also obtained under a water-based environment. The significant cell migration and proliferation in the wound healing assay were observed, indicating the excellent ability of O_2_ and NO supply in cell activation. Moreover, the Mn-p-MOF-NO showed superior efficacy in wound repair and reassuring biosafety in mice’s acute wound model, indicating a breakthrough development in wound treatment by dual gas therapy using a MOF-based nanocarrier.

## Supplementary Material



## Notes

Figure S1: the flow diagram of the NO loading process for Mn-p-MOF-NO preparation; Figure S2: XPS analysis of the Mn-p-MOF nanozymes in dimethylformamide (DMF) and H_2_O; Figure S3: the high-resolution TEM images of nanozymes (a) before and (b) after NO loading; Figure S4: the dark-field high-resolution TEM images of nanozymes (a) before and (b) after NO loading; Figure S5: the cellular HIF-1α expression of NIH/3T3 cells treated with Mn-p-MOFNO nanozymes at 1 ppm of Fe element; Figure S6: the binding energies of C_1S_, O_1S_, and N_1S_ of Mn-p-MOF nanozymes in (a) dimethylformamide and (b) H_2_O; and Figure S7: the biodistribution analysis for mice treated with and without Mn-p-MOFNO nanozymes at day 0. (**P* < 0.05; n.s.= no significance) (PDF)
