# Supplementary material for: Water-Driven Oxygen and Nitric Oxide Release from Porphyrin- and Mn-Based Metal–Organic Framework Enables Accelerated Acute Wound Healing
Source: ACS Appl Bio Mater. 2025 Sep 1;8(9):7616–27. doi: 10.1021/acsabm.5c00135 (PMC12442097; doi:10.1021/acsabm.5c00135)
Supplement: Supplementary file 1 [file mt5c00135_si_001.pdf]

Supporting Information

Water-Driven Oxygen and Nitric Oxide Release from Porphyrin and Mn-Based Metal-Organic Framework Enables Accelerated Acute Wound Healing

Jieh-Neng Wang[1], Zih-Yu Tu[2], Wen-Jyun Wang[2], Wei-Peng Li[2,3,4,5,], Wei-Ling Chen[6,7,]*, and Chung-Dann Kan[8,]**

[1]Department of Pediatrics, National Cheng Kung University Hospital, College of Medicine, National Cheng Kung University, Tainan 704, Taiwan

[2]Department of Medicinal and Applied Chemistry, Kaohsiung Medical University, Kaohsiung 807, Taiwan

[3]Department of Medical Research, Kaohsiung Medical University Hospital, Kaohsiung 807, Taiwan

[4]Drug Development and Value Creation Research Center, Kaohsiung Medical University, Kaohsiung 807, Taiwan

[5]Center of Applied Nanomedicine, National Cheng Kung University, Tainan 701, Taiwan

[6]Department of Biomedical Engineering, Taipei Veterans General Hospital, Taipei 112, Taiwan

[7]Department of Biomedical Engineering, National Yang Ming Chiao Tung University, Taipei 112, Taiwan.

[8]Division of Cardiovascular Surgery, Department of Surgery, National Cheng Kung University Hospital, College of Medicine, National Cheng Kung University, Tainan 704, Taiwan

*Corresponding Email: wpli@kmu.edu,tw; lynnchen.k@gmail.com; kcd56@mail.ncku.edu.tw

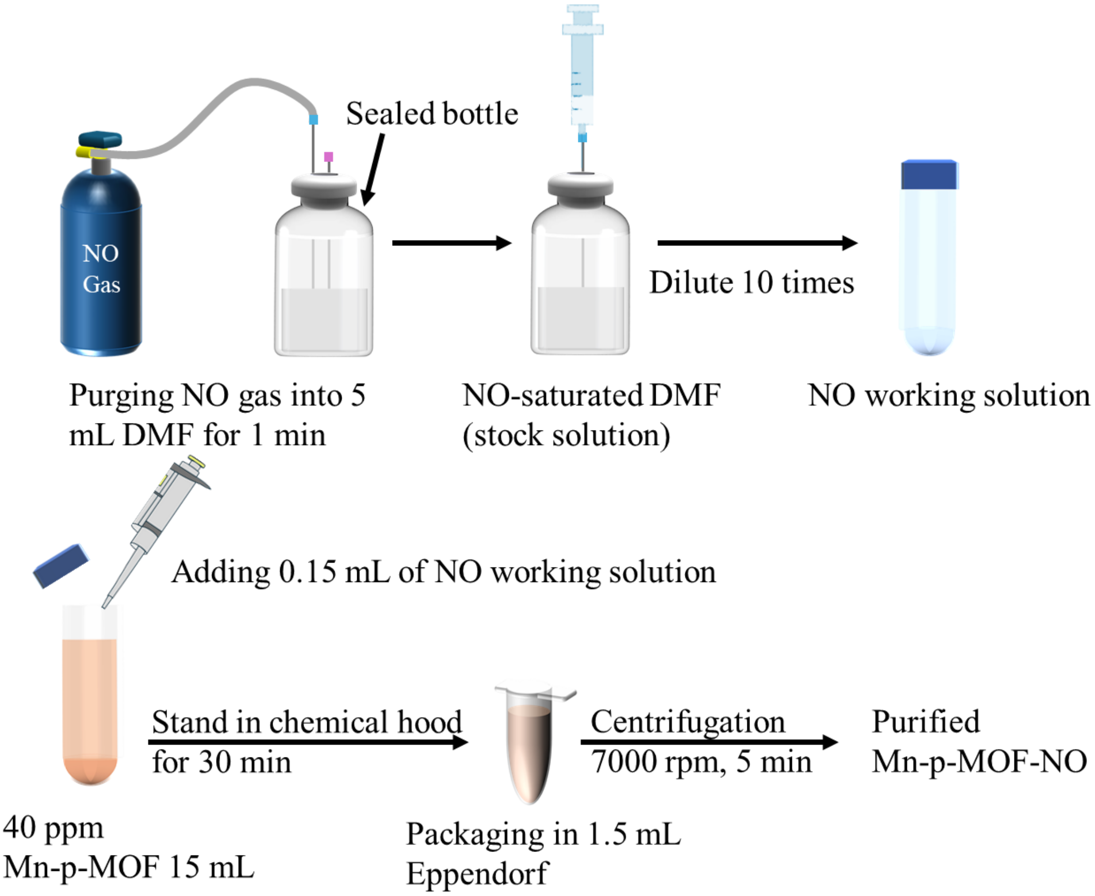

Figure S1. The flow diagram of the NO loading process for Mn-p-MOF-NO preparation.

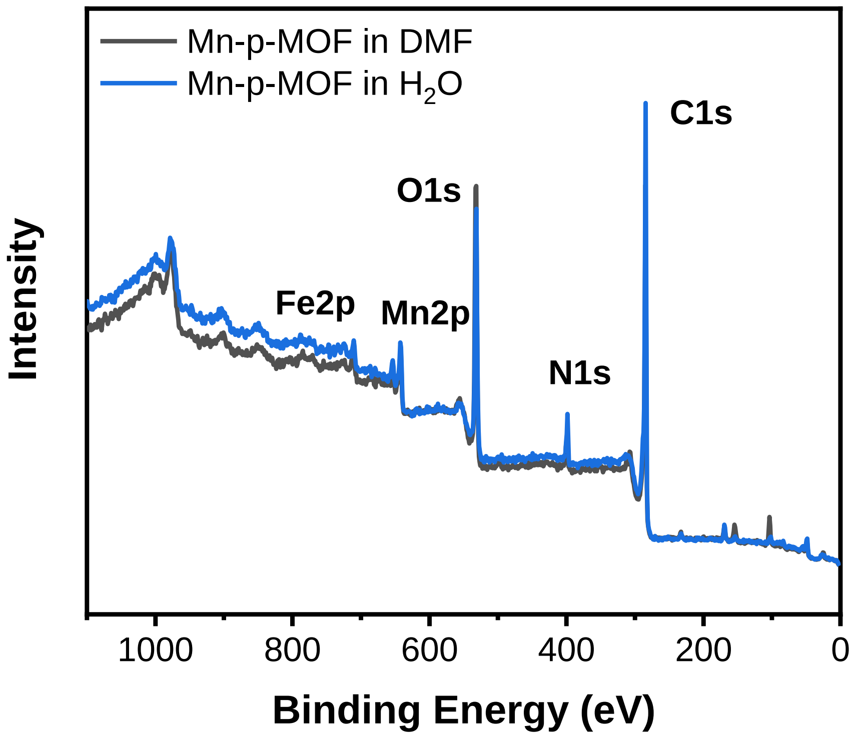

Figure S2. XPS analysis of the Mn-p-MOF nanozymes in dimethylformamide (DMF) and H_2O.

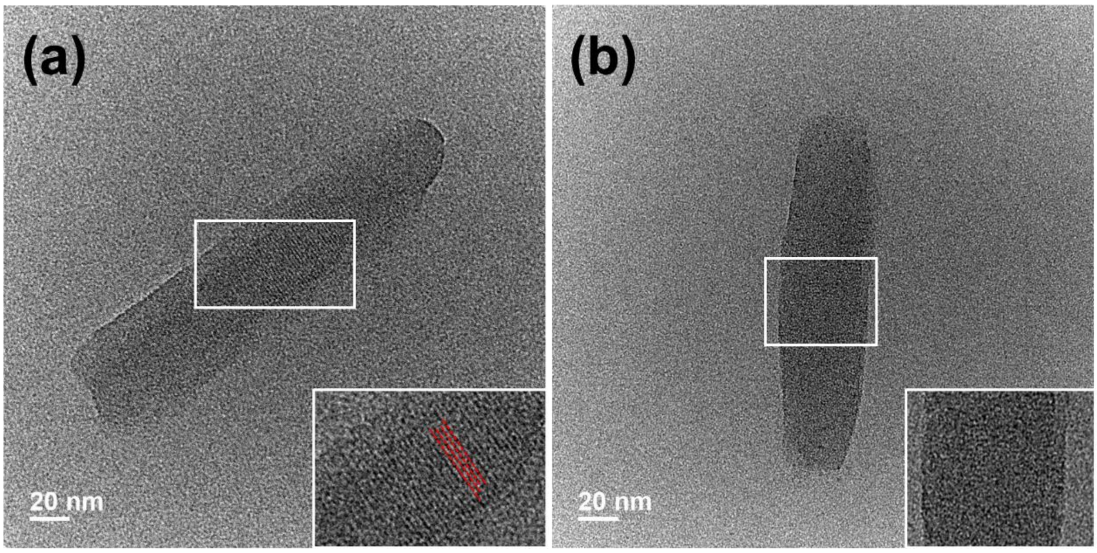

Figure S3. The high-resolution TEM images of nanozymes (a) before and (b) after NO loading. The inserted illustrations showed the amplified areas labeled by the white frame. The red lines in the insert image indicated the [110] lattice plane in the spindle-shaped Mn-p-MOF nanozyme.

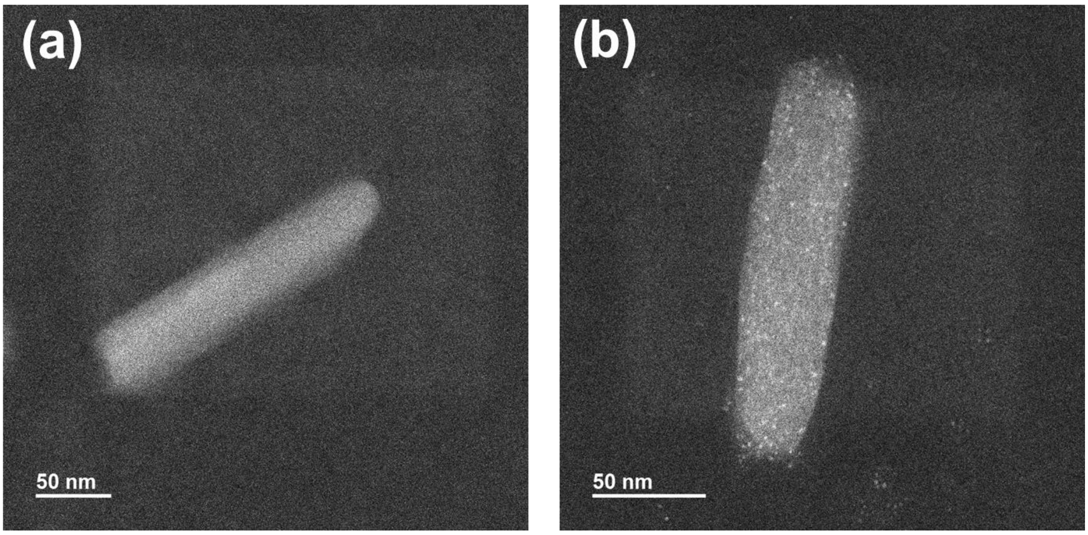

Figure S4. The dark-field high-resolution TEM images of nanozymes (a) before and (b) after NO loading.

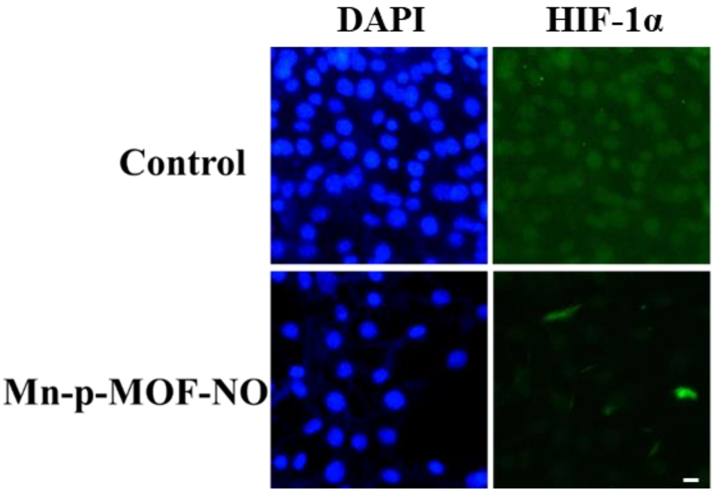

Figure S5. The cellular HIF-1α expression of NIH/3T3 cells treated with Mn-p-MOF-NO nanozymes at 1 ppm of Fe element. The nucleus and HIF-1α were stained by DAPI and HIF-1α antibody, respectively. The scale bar is 10 μm.

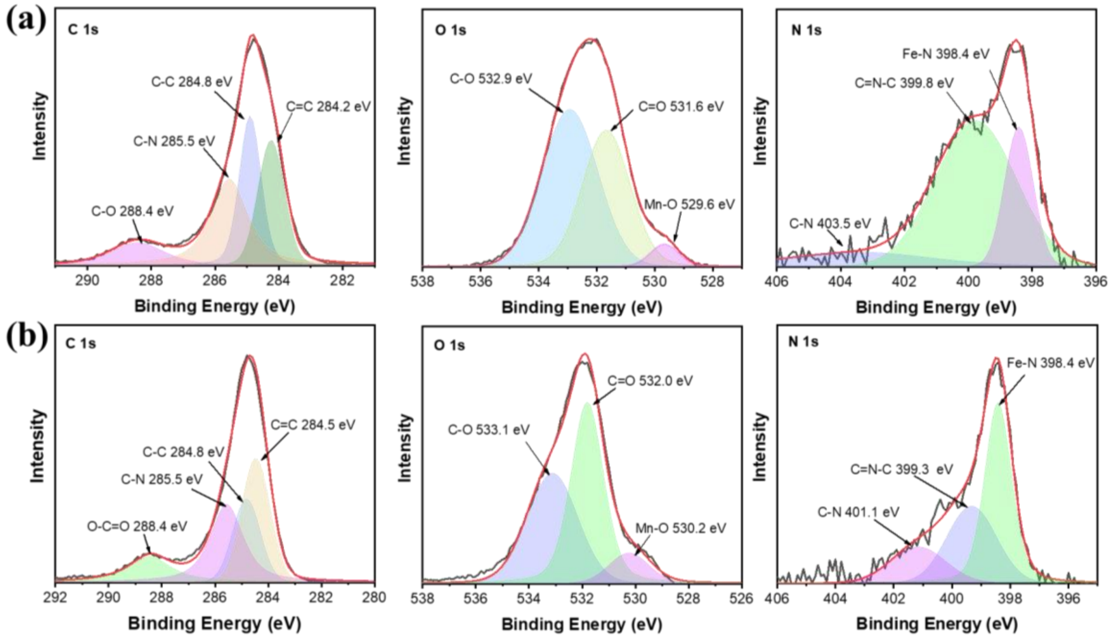

Figure S6. The binding energies of C_{1S}, O_{1S}, and N_{1S} of Mn-p-MOF nanozymes in (a) dimethylformamide and (b) H_2O.

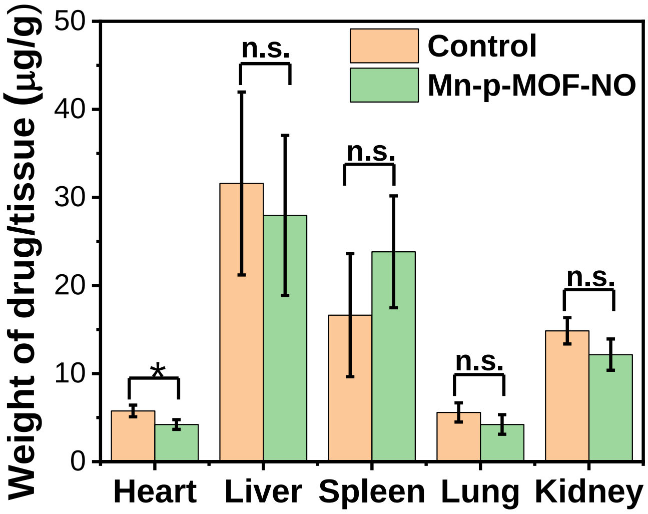

Figure S7. The biodistribution analysis for mice treated with and without Mn-p-MOF-NO nanozymes at day 0. (*P < 0.05; n.s.= no significance)
